# *Musa paradisiaca* L. Inflorescence Abrogates Neutrophil Activation by Downregulating TLR4/NF-KB Signaling Pathway in LPS-Induced Acute Lung Injury Model

**DOI:** 10.3390/ph18010008

**Published:** 2024-12-24

**Authors:** Francisco Allysson Assis Ferreira Gadelha, Raquel Fragoso Pereira Cavalcanti, Cosmo Isaias Duvirgens Vieira, Joao Batista De Oliveira, Louíse Mangueira De Lima, Adriano Francisco Alves, Matheus Marley Bezerra Pessoa, Leônia Maria Batista, Naiara Naiana Dejani, Marcia Regina Piuvezam

**Affiliations:** 1 Laboratory of Immunopharmacology, Research Institute for Drugs and Medicines, Federal University of Paraíba, João Pessoa 58051-900, PB, Brazil; allyssongadelha@ltf.ufpb.br; 2 Postgraduate Program in Natural and Synthetic Bioactive Products, Federal University of Paraíba, João Pessoa 58051-900, PB, Brazil; raquelfragoso@ltf.ufpb.br (R.F.P.C.); cosmoisaiasv@gmail.com (C.I.D.V.); joa1.oliv@gmail.com (J.B.D.O.); louisemangueira123@gmail.com (L.M.D.L.); mmarleybp@gmail.com (M.M.B.P.); leoniabatista1@gmail.com (L.M.B.); 3 Department of Phisiology and Phatology, Health Sciences Center, Federal University of Paraíba, João Pessoa 58051-900, PB, Brazil; adrianofalves@gmail.com (A.F.A.); naiaradejani@gmail.com (N.N.D.)

**Keywords:** banana heart, immunomodulatory activity, DNA, myeloperoxidase

## Abstract

**Background/Objectives**: Acute lung injury (ALI) is an inflammatory disorder affecting patients in intensive care with high mortality. No specific pharmacological treatment is available. *Musa paradisiaca* L. (banana) is a cosmopolitan plant, and homemade syrup from its inflorescence is used in many countries to treat pulmonary inflammation. Therefore, this study analyzed the hydroalcoholic extract (HEM) of the inflorescence on the ALI experimental model. **Methods**: Swiss mice were challenged with lipopolysaccharide and treated with HEM after 1, 24, and 48 h (five animals/group, three times). **Results**: The HEM-treated ALI mice presented a decrease in neutrophil migration in the bronchoalveolar lavage fluid (BALF), in the alveolar region, and in the blood, correlating to downregulation of CD18 expression. The HEM treatment also reduced the protein concentration in the BALF, caused lung edema formation, impaired NF-κB activation via inhibition of TLR4 signaling pathway, and decreased IL-1β, TNF-α production, free DNA release, and myeloperoxidase (MPO) activity. However, the extract induced an increased IL-10 in the BALF. **Conclusions**: Therefore, HEM’s anti-inflammatory and immunomodulatory activities in ALI mice are by deactivating neutrophils by decreasing CD18 receptor, free DNA release, and MPO activity and inducing IL-10 production. Thus, this study supports the use of banana inflorescence in folk medicine and suggests its rational use to develop a phytomedicine to treat pulmonary inflammation.

## 1. Introduction

Acute lung injury (ALI) and its more severe form, acute respiratory distress syndrome (ARDS), are human pulmonary inflammatory conditions characterized by neutrophil infiltration into the lungs, disruption of the epithelial barrier of the pulmonary endothelium, loss of alveolar-capillary membrane integrity, and reduction in lung compliance [1]. The mortality rate of patients with ALI/ARDS is about 40–58% and is associated with multiple organ dysfunction [2]. The physiopathology of ALI/ARDS involves cytokine production promoting a biological condition called “cytokine storm” and an immune-cell overactivation, a phenomenon well described in severe COVID-19 syndrome [3].

Neutrophils are considered to play a key role in the progression of ALI, and neutrophil recruitment into the lung has been considered a hallmark of ALI [4]. In the model of ALI, LPS engages the TLR4/NF-kB pathway inducing inflammation and neutrophil recruitment to the lungs, activating them to produce reactive oxygen species, increase the expression of adhesion proteins such as integrin (CD18), as well as the production of cytokines, such as TNF-a, IL-1β, IL-6, and the release neutrophil extracellular traps (NETs—free DNA, myeloperoxidase, histone), promoting diffuse alveolar damage and, consequently, difficulty in gas exchange [5]. ARDS/ALI has no specific treatment, and the current drugs do not reduce morbidity and mortality [6]. Therefore, the goal of medicines to treat ALI must be a reduction in lung inflammation and respiratory arrest inhibition.

*Musa paradisiaca* L., popularly named banana, is among the main crops in the world and is food mainly for the people of tropical countries [7]. Brazil is the fourth world producer of bananas, with approximately six thousand tonnes in 2021 [8]. Many parts of the plant are used based on their medicinal properties, which include powerful antioxidant, anti-ulcerogenic, anti-microbial, hypoglycaemic, hepatoprotective, and anti-tumor activity [9,10].

In Brazil, the inflorescence of *Musa paradisiaca* L. is popularly known as the “banana heart” and “mangará”, and chemical analysis has shown the presence of polyphenols such as gallic acid, catechol, caffeic acid, ferulic acid, p-coumarinic acid, and compounds such as myricetin, kaempferol, apigenin, lupenone, pentacosane, and 10-hentriacontene [11,12]. However, in large-scale production, the inflorescence of *Musa paradisiaca* L. is used as a fertilizer, with values that reach one thousand tons per year [8]. In many countries such as India, Malaysia, Taiwan, Srilanka, Indonesia, South America, and Brazil, the inflorescence is used as food and homemade medicine to treat inflammatory diseases of the respiratory tract such as asthma, bronchitis, and tuberculosis [13,14] becoming an attractive plant tool to treat illness.

In a previous study, the *Musa paradisiaca* L. inflorescence extract (HEM) showed an immunomodulatory activity in a combined allergic rhinitis and asthma syndrome (CARAS) model. The HEM decreased the inflammatory cell migration to the respiratory tract and type 2 cytokines, and inhibited the NF-kB signaling pathway in eosinophils [14].

Therefore, the present study aims to demonstrate the HEM effect in a lipopolysaccharide-induced acute lung injury to add scientific knowledge to the plant material and, therefore, cease to be an agricultural disposable for treating pulmonary illness.

## 2. Results

### 2.1. HEM Improves Animal Survival and Reduces Inflammatory Cell Migration in the BALF in LPS-Induced ALI

**Figure 1:**
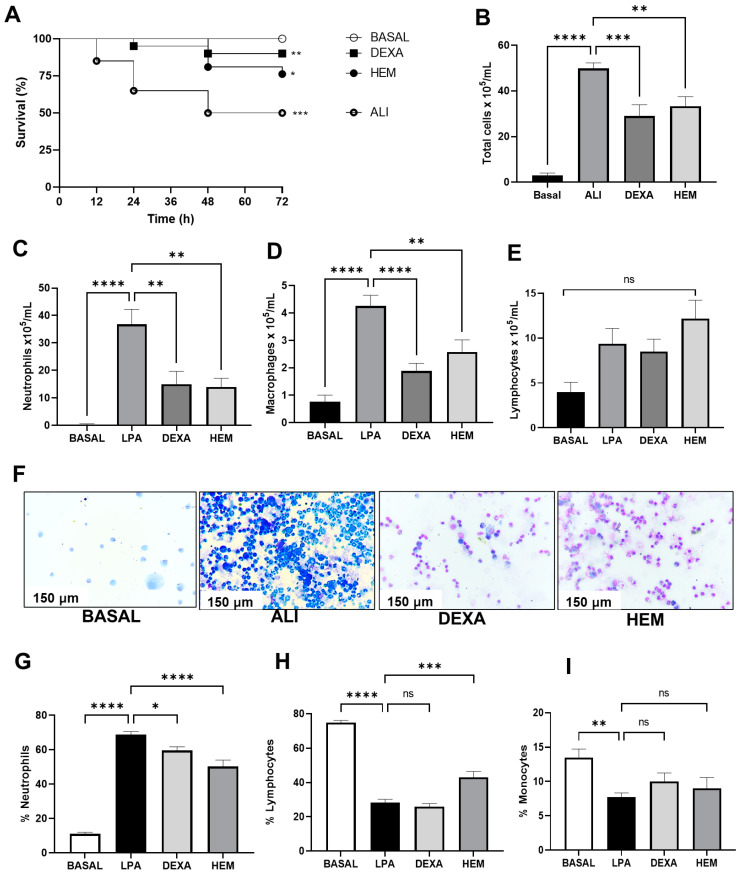
HEM improves animal survival and decreases neutrophilic inflammation in LPS-induced ALI. The animal survival was quantified during the 72 h of LPS-challenged (**A**). The total cell number in the BALF was quantified in the Newbauer camera (**B**). Differential cells were counted in the BALF (**C**–**E**), and in the blood (**G**–**I**) by optical microscopy. Photomicrographs are representative of the stained cells of the cytocentrifuged procedure (**F**). The data are presented as ± SEM. Data were analyzed using one-way ANOVA followed by Bonferroni post-test. * *p* < 0.05, ** *p* < 0.005, *** *p* < 0.001 or **** *p* < 0.0001. BASAL (no LPS-challenged), ALI (LPS-challenged), DEXA (LPS-challenged and orally treated with dexamethasone), HEM (LPS-challenged and orally treated with HEM).

The ALI group had a survival rate of 50% compared to the BASAL group, while HEM treatment increased the animal survival rate by 25% (Figure 1A). HEM also significantly decreased total cell, neutrophil, and macrophage migration in the BALF, while the number of lymphocytes was unaffected (Figure 1B–F). A similar effect was seen on the DEXA group (Figure 1B–F). In the blood of ALI mice, there was an increase in the percentage of neutrophils that was decreased with the HEM treatment and an increase in lymphocytes without affecting monocyte percentage (Figure 1G–I). Dexamethasone treatment decreased the percentage of neutrophils, without altering lymphocytes and monocytes (Figure 1G–I).

### 2.2. HEM Decreases Pulmonary Edema and Inflammation in LPS-Induced ALI

**Figure 2:**
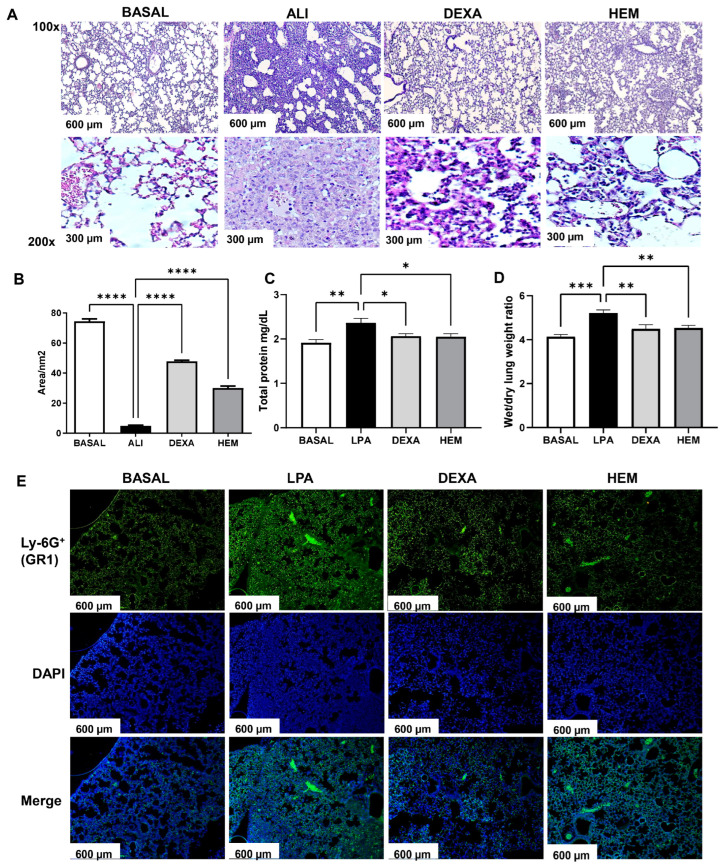
HEM reduces edema and neutrophilic inflammation in the ALI model. Histological analysis (**A**,**B**), protein exudate in the BALF (**C**), lung wet/dry weight ratio (**D**), and lung immunofluorescence (**E**) (magnification: 100×). Protein was quantified in the BALF, and the lung was used to determine the weight/dry ratio and morphometric and immunofluorescence analysis. For immunofluorescence analysis, neutrophils in lung tissue were labeled with anti-Ly-6G (GR1 + cells) and DAPI. The photomicrographs of histology and immunofluorescence procedures are representative of five animals per group. The data are presented as ± SEM. Data were analyzed using one-way ANOVA followed by Bonferroni post-test. * *p* < 0.05, ** *p* < 0.005, *** *p* < 0.001 or **** *p* < 0.0001. BASAL (no LPS-challenged), ALI (LPS-challenged), DEXA (LPS-challenged and orally treated with dexamethasone), HEM (LPS-challenged and orally treated with HEM).

The BASAL group presented preserved alveolar architecture; the ALI group showed an intense cell migration, and the presence of edema and hemorrhage, characterized by a decrease in the free alveolar area (Figure 2A). HEM or dexamethasone treatments reduced these signs of inflammation and maintained a large free alveolar area (Figure 2A,B). HEM and DEXA groups also showed a reduction in protein concentration in the BALF and the lung wet-to-dry weight ratio compared to the ALI group (Figure 2C,D). The immunofluorescence evaluation confirmed that neutrophils are the prior cells in the lung tissue of animals of the ALI group due to high staining for Ly6G (GR1 + neutrophils) (Figure 2E). HEM treatment decreased staining for Ly6G (GR1 + cells) in the pulmonary tissue (Figure 2E).

### 2.3. HEM Impairs NF-κB Phosphorylation and Cytokine Production and Decreases TLR4 and CD18 Expression in Cells of the BALF in LPS-Induced ALI

**Figure 3:**
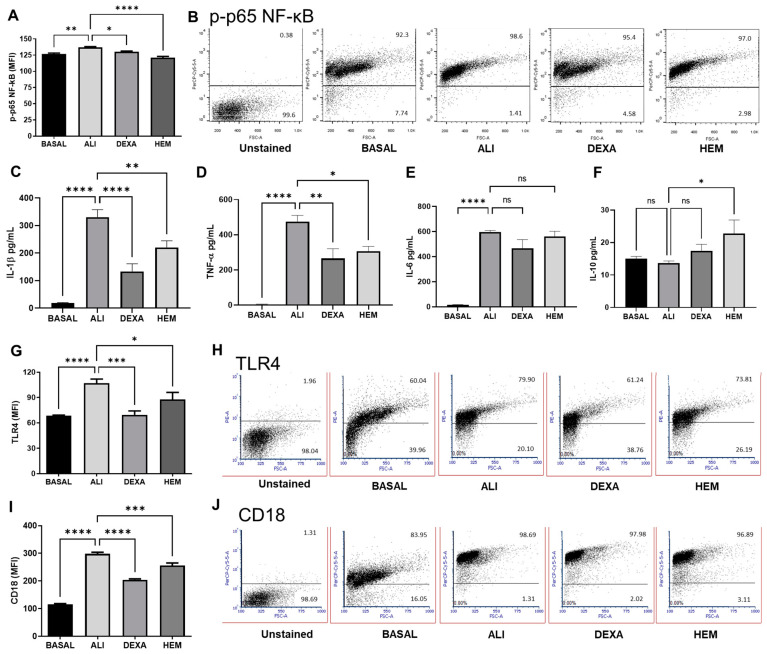
HEM impairs NF-κB activation, cytokines production and TLR4 and CD18 expression in BALF cells of ALI mice. p-p65 MFI values (**A**), percentage of p-p65 positive cells (**B**), IL-1β, TNF-α, IL-6 and IL-10 cytokine quantification (**C**–**F**), TLR4 MFI (**G**) and percentage of TLR4 positive cells (**H**), and CD18 MFI (**I**) and percentage of CD18 positive cells (**J**) from the BALF of animals. The cells were stained with anti-p-p65-NF-κB, anti-TLR4 or anti-CD18, and at least 10,000 events were evaluated. Cytokine concentration was determined in the BALF by ELISA assay. The data are presented as ± SEM. Data were analyzed using one-way ANOVA followed by Bonferroni post-test. * *p* < 0.05, ** *p* < 0.005, *** *p* < 0.001 or **** *p* < 0.0001. BASAL (no LPS-challenged), ALI (LPS-challenged), DEXA (LPS-challenged and orally treated with dexamethasone), HEM (LPS-challenged and orally treated with HEM).

The inflammatory response in ALI depends on the phosphorylation and translocation of the nuclear factor kappa B (NF-κB) to the nucleus to induce pro-inflammatory cytokines, chemokines, and receptor expression either in circulating or tissue immune cells. The phosphorylated form (p-p65) of NF-κB expressed as the mean fluorescent intensity (MFI) was higher in BALF cells of the ALI group compared to the BASAL group (Figure 3A), while the percentage of the cells was unchanged (Figure 3B). HEM or DEXA treatments significantly decreased the p-p65 NF-κB MFI without affecting the percentage of p-p65 positive cells (Figure 3A,B). In addition, these treatments reduced IL-1β and TNF-α levels in the BALF (Figure 3C,D). However, neither HEM nor dexamethasone treatments decreased IL-6 production (Figure 3E). Surprisingly, IL-10 levels were higher in the HEM group compared to the ALI group (Figure 3F). The over-expression of TLR4 and CD18 in BALF cells of the ALI group was observed. Indeed, the TLR4 and CD18 MFI in these cells were high compared to the BASAL group, whereas the treatments with HEM or DEXA significantly reduced both markers (Figure 3G–J).

### 2.4. HEM Downregulates Neutrophil Activity in LPS-Induced ALI Mice Without Affecting Cell Viability

**Figure 4:**
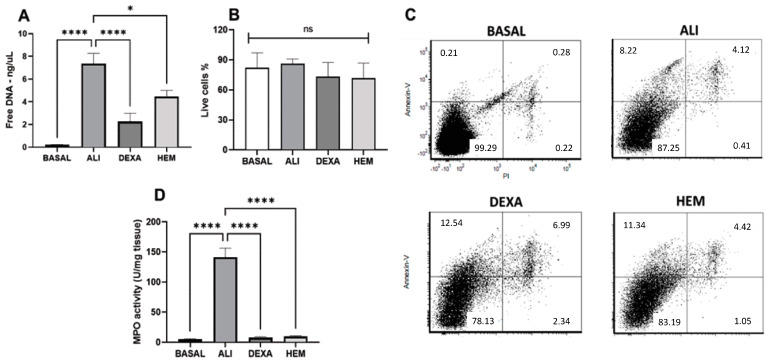
HEM reduces free DNA and MPO activity without affecting neutrophil death in ALI mice. Quantification of free DNA in the BALF (**A**), percentage of live cells in the BALF by annexin V and PI staining (**B**), representative flow cytometric plots (**C**), and mieloperoxidase activity (**D**). The data are presented as ± SEM. Data were analyzed using one-way ANOVA followed by Bonferroni post-test. * *p* < 0.05, or **** *p* < 0.0001. BASAL (no LPS-challenged), ALI (LPS-challenged), DEXA (LPS-challenged and orally treated with dexamethasone), HEM (LPS-challenged and orally treated with HEM).

The ALI group showed a significant increase in the free DNA in the BALF compared to the BASAL group (Figure 4A), whereas HEM or DEXA groups presented a reduction in the amount of free DNA in the BALF (Figure 4A). The analysis of the percentage of annexin V and PI negative cells pre-gated on a granulocytic population (neutrophil), which represents about 90% of total cells in BALF, showed no significant difference among the groups (Figure 4B,C). In addition, MPO activity in the lung tissue was significantly higher in the ALI group than in the BASAL group. On the other hand, the HEM or DEXA groups presented decreased MPO enzyme activity compared to the ALI group (Figure 4D).

## 3. Discussion

The inflorescence of *Musa paradisiaca* L. is used in several countries including Brazil, for therapeutic purposes on digestive and respiratory tract disorders [7,10]. However, tons of the inflorescence are disposed of as fertilizer in large banana crops [8]. Thus, some research groups have tried to scientifically support the inflorescence to make better use of this plant in the production of phytomedicine to treat inflammation.

Our group has studied the *Musa paradisiaca* L. inflorescence hydroalcoholic extract (HEM) in an experimental model of combined asthma and allergic rhinitis syndrome (CARAS) and demonstrated that the extract inhibited the eosinophil migration to the lung tissue and decreased the Th2 immune response towards a Treg immune response. In addition, the HEM was fractionated into four parts according to the solvents: ethyl acetate, dichloromethane, N-butanol, and hexane, and, from the chemical analysis of the ethyl acetate and N-butanol fractions, we previously identified the presence of the following compounds: quercetin, myricetin, rutin, gallic acid, protocatechuic acid, ethyl gallate, quinic acid, 4-O-caffeoylshikimic acid I, hydroxycinnamic acid and 1-Sinapoyl-2-feruloylguthiobiose [14].

Herein, in this study, we evidenced the anti-inflammatory and immunomodulatory effects of HEM on the LPS-induced acute lung injury (ALI) model. HEM-treated ALI mice presented a reduction in neutrophils in the lung and blood. HEM also decreased alveolar edema, reduced BALF cell expression of TLR4 and CD18, and inhibited IL-1β and TNF-α production, while enhancing IL-10 levels in the BALF. These findings were associated with downregulation of NF-κB activation, MPO activity, and free DNA levels in lung tissues. The immunomodulatory effect produced by HEM was a determinant factor in increasing animal survival, from 50% to 75%.

The LPS-induced ALI mice model is characterized by intense neutrophil lung infiltration in the first hours of the disease [15]. These cells act in the development of the inflammatory process and are the effector cells in ALI [16]. Neutrophils are also responsible for the aggressiveness of lung injury via the release of proteolytic enzymes, oxygen and nitrogen species, and NETs [5]. We demonstrated that oral HEM-treated ALI mice decreased neutrophils in the BALF and blood, characterizing a local and systemic modulation. Indeed, the HEM treatment reduced the cell CD18 expression, an adhesion molecule of the leukocyte migration process. Thus, this effect may be related to the presence of the immunomodulatory compounds in the extract as quercetin, rutin, and ethyl gallate, which have been described as protective against oxidative stress and cellular infiltration in the lungs of ALI mice [5,17,18].

Alveolar edema is one of the most remarkable parameters in ALI, and it is closely related to the mortality of affected individuals [19]. Increased vascular permeability facilitates cell migration, and protein exudate in the lung cavity worsening gas exchange [20]. The evaluation of the lung wet/dry weight ratio, which is associated with protein exudate in the BALF, demonstrated significant anti-edematogenic activity of the HEM. Some of the compounds in the HEM such as protocatechuic acid, kaempferol, and quercetin have been described to reduce the protein exudate in the BALF, lung wet/dry weight ratio, and vascular extravasation in an ALI model corroborating our results [21,22]. Complementary data came from the histological analysis that demonstrated an inflammatory pattern in ALI mice as alveolar edema, intense lung cell infiltration, and hemorrhagic points, and the HEM treatment drastically reduced these inflammatory parameters.

In the inflammatory process, immune cell communication occurs through cytokines, e.g., increases in IL-1β in the lungs of ALI animals induce lung epithelium permeability with tissue damage. In addition, IL-6 and TNF-α production are related to lung neutrophil recruitment and worse outcomes in patients with ALI [6]. Therefore, BALF of HEM-treated ALI mice presented a reduction in IL-1β, and TNF-α independently of IL-6. These contradictory data may be due to the difference between the chronic diabetes model and the acute lung injury model and the fact that, in the ALI model, the treatment was made with the inflorescence banana crude extract instead of the inflorescence ethyl acetate fraction [9]. These contradictory data may also be due to differences in the plant material and the experimental models.

In addition, HEM-treated ALI mice showed an enhancement of IL-10 levels in the BALF. IL-10 is an anti-inflammatory cytokine, and its production is increased in several diseases, including pulmonary illnesses such as ALI and asthma in an attempt to reestablish homeostasis [23]. Thereby, the upregulation of IL-10 production in the ALI model by the HEM is promising evidence of its anti-inflammatory and immunomodulatory properties. This HEM effect was previously reported in the ovalbumin-induced CARAS experimental model, where the upregulation of IL-10 production decreased allergic lung inflammation [14] showing that the anti-inflammatory effect of HEM may occur independently of the type of inflammation stimulus.

The LPS-induced ALI model is related to the TLR4 activation, which induces signaling pathways to promote pro-inflammatory cytokine production and several other inflammatory molecules [24]. Therefore, BALF cells expressing TLR4 were significantly enhanced in ALI mice, and HEM treatment decreased the number and the mean fluorescence intensity (MFI) of TLR4+ cells. Shokry and co-workers demonstrated in an ALI model the downregulation of TLR4 expression and reduction in oxidative stress by a fraction of *Hedera helix* L. rich in phenolic compounds that present anti-inflammatory properties [25]. Therefore, HEM is rich in such compounds; thus, the synergist action of these compounds generates the beneficial immunomodulatory effect in LPS-induced ALI.

The LPS-activated-TLR4 signaling pathway induces the production of inflammatory cytokines by the phosphorylated p-p65 NF-κB translocated to the nucleus [24]. This phenomenon happens in LPS-induced ALI [26]. Therefore, evaluating the p-p65 NF-κB, we observed a reduction in the phosphorylation process in BALF cells of ALI mice treated with HEM. In addition, the reduction in NF-κB activation by HEM was related to the decrease in IL-1β and TNF-α production as described above. These data were corroborated by a recent study that showed the effect of myricetin, a phenolic compound, modulating lung inflammation in an ALI model by inhibiting NF-κB activation and inflammatory cytokine production [27].

The neutrophil accumulation in the lung tissue of ALI mice and the release of NETs, which are composed of free DNA complexes and enzymes, such as MPO, are related to pathogenic mechanisms in ALI, including lung damage and inflammation [28]. In our study, ALI mice presented a high amount of free DNA and a high MPO activity in the BALF, while HEM treatment decreased both parameters. Therefore, the reduction in the release of these molecules by the HEM treatment indicates its protective effect by decreasing the neutrophil activation and thus ameliorates the lung tissue in an ALI condition.

## 4. Material and Methods

### 4.1. Animals

Female Swiss mice (6–8 weeks old, weighing 27 ± 2 g) were used for the acute lung injury (ALI) development. Three independent sets of experiments were performed using five animals per group. The animals were kept in polypropylene cages at 21 ± 1 °C and were subjected to 12 h light/dark cycles with free access to water and food throughout the experimentation period. The experimental procedures were approved by the Ethical Committee from the UFPB, Brazil (protocol no. 7316150420 (ID 001064).

### 4.2. Hydroalcoholic Extract of Musa paradisiaca L. Inflorescence

The hydroalcoholic extract of *M. paradisiaca* L. inflorescence (HEM) was obtained at the Phytochemical Laboratory of UFPB, Paraíba, Brazil, by using a percolator-type macerator and the compounds are quercetin, myricetin, rutin, gallic acid, protocatechuic acid, ethyl gallate, quinic acid, 4-O-caffeoyl shikimic acid I, and hydroxycinnamic acid (HPLC-MS/MS) (Supplementary Materials). The inflorescence was harvested in domestic crops (7008′14.6″ S 34052′26.1″ W), Paraíba, Brazil. The toxicity assays were carried out following the OECD 423/2001 guidelines [29], and the HEM presented LD50 ≥ 5000 mg/kg without genotoxicity or psychosomatic alterations [14]. Based on our previous data, the dose used in this study was 100 mg/kg [14].

### 4.3. Murine Model of Lipopolysaccharide (LPS)-Induced Acute Lung Injury (ALI)

Swiss mice (*N* = 5 per group) were anesthetized and received, by nasal instillation, 40 μL of LPS solution (5 mg/kg, LPS of Escherichia coli O111:B4—Sigma-Aldrich^®^, St. Louis, MO, USA, 048 M4064V) [30]. The animal groups were defined as BASAL (healthy, no LPS-challenged), ALI (LPS-challenged non-treated), HEM (LPS-challenged and orally treated with HEM at 100 mg/kg), and DEXA (LPS-challenged and orally treated with dexamethasone at 2 mg/kg—Ache^®^, Sao Paulo, SP, Brazil). The animals were treated 1, 24, and 48 h after the LPS challenge. After 72 h, the animals were euthanized by xylazine at 1.91 mg/mL and ketamine at 29 mg/mL.

### 4.4. Animal Survival Analysis

Animals were observed for 72 h, and live animals were counted every 12 h. After this period, the survival percentage was defined [15].

### 4.5. Inflammatory Cells in Bronchoalveolar Lavage Fluids (BALFs) and Blood

BALF and blood were collected 72 h after the LPS challenge. An intratracheal cannula was administered to the lung with 1.5 mL of PBS, and the BALF was harvested. The Neubauer chamber determined the number of total cells under an optical microscope (40×—BX40, OLYMPUS, Tokyo, Japan). Cytocentrifuged BALF slides (FANEN, São Paulo, SP, Brazil, Mod 2400) and blood smear were stained (Panoptic Kit, Renylab, Barbacena, Brazil) for differential cell count.

### 4.6. Protein Concentration in the BALF

Total protein in BALF was quantified by colorimetric method using pyrogallol dye, according to the manufacturer’s instructions (Sensiprot kit REF 36—1804—LABTEST, Vista Alegre, Lagoa Santa, Brazil) [20].

### 4.7. Cytokine Quantification in the BALF

IL-1β (REF 88-7013-88), IL-6 (REF 88-7064-88), TNF-α (REF 88-7324-88) (Invitrogen Thermo Fisher Scientific, Viena, Austria), and IL-10 (REF 14-7102-68, EBIOSCIENCE, San Diego, CA, USA) were quantified by ELISA, according to manufacturer’s instructions.

### 4.8. Cell Analysis by Flow Cytometry

The BALF cells (2.5 × 10^5^) were fixed, permeabilized, and labeled with anti-CD18 (BD PercCP-Cy5.5 Anti-Mouse CD18, Cat# 562827), anti-TLR4 (eBioscience, San Diego, CA, USA, PE anti-mouse CD284 (TLR4), Cat# 12-9041-80) or anti-p-p65 NF-κB, according to the manufacturer’s instructions (BD Phosflow PE, Cat# 558,423). Ten thousand events were analyzed by flow cytometry (BD FACSCANTO II) and FlowJo V.10 software.

### 4.9. Histological Analysis

The right lung of each animal was processed in alcohol, xylol, and paraffin baths. Slides with lung tissues were rehydrated and stained with hematoxylin-eosin, and cellular infiltrate, hemorrhage, and alveolar edema were defined. The representative photomicrographs were analyzed using Motic Images Plus 2.0 software. Histological score/morphometry was analyzed, and the intensity of inflammation is related to the filling of lung tissue (alveolar region) and quantified by morphometric analysis expressed as the area of free alveolar space (area/nm^2^) of tissue [31].

### 4.10. Wet Weight/Dry Weight Lung Ratio

The lung was weighed to determine the wet weight, placed in a drying oven at 60 °C for 48 h, and weighed to determine the dry weight. The pulmonary edema index was calculated through the wet/dry weight ratio of each lung [20].

### 4.11. Free DNA Quantification

The free DNA quantification in the BALF was performed following the instructions of the dsDNA HS Assay Kit manufacturer (Cat# Q32854 Invitrogen, ThermoFisher Scientific, Life Technologies Corporation, Eugene, OR, USA). BALF samples were diluted in the reagent mix (1:200), and after 5 min, they were analyzed in the Qubit 4 Fluorometer—Invitrogen.

### 4.12. Cell Death

Cell death in the BALF was assessed by flow cytometry using annexin V (eBioscience REF11-8005-74 FITC) and propidium iodide (PI) (eBioscience REF 00-6990-42), according to the manufacturer’s protocol. The percentage of dying cells was analyzed in FlowJo software.

### 4.13. Immunofluorescence in Lung Tissue

Lung tissue was deparaffinized twice in xylene for 3 min, once in xylene/ethanol 100% for 3 min and ethanol 100%, 95%, 70%, and 50% for 3 min. The slides with the lung tissue were washed twice for 5 min in PBS with 0.025% Triton X-100 and blocked in 2% BSA for 2 h. Then, slides were incubated with anti-Ly6G (GR1) (eBioscience FITC CAT# 11-5931-82) and mounted in DAPI Fluoromount-G (REF 0100-20 Southern Biotech, Birmingham, AL, USA). A digital camera MOTIC 5.0 MP coupled to a BA410 fluorescence microscope (Motic BA410) was used to capture images [6].

### 4.14. Myeloperoxidase (MPO) Activity in Lung Tissue

MPO activity was measured and expressed as units per gram of tissue, as described by [32].

### 4.15. Statistical Analysis

GraphPad Prism software version 9.0 (San Diego, CA, USA) was used to perform statistical analyses. The non-parametric one-way analysis of variance (ANOVA) method followed by Tukey’s or Bonferroni post-test for multiple comparisons was used. To analyze the results of histological scores, Kruskal–Wallis test was used. The *p* < 0.05 was considered statistically significant and expressed as mean ± standard error of the mean (SEM).

## 5. Conclusions

The banana inflorescence crude extract treatment of animals with acute lung injury increased animal survival by downregulating the lung neutrophil migration, decreasing the inflammatory cytokine production as IL-1B and TNF-a, the TLR4 and CD18 expression and NF-kB activation. Also, the extract downmodulated the NETs’ release and MPO activity. Therefore, the extract decreased some of the most relevant inflammatory parameters in the ALI experimental model, putting it as a potential material to be the base of a pharmaceutical product, thus offering a sustainable destination to the inflorescence routinely disposed of on Brazilian banana crops.

## Data Availability

Data is contained within the article.

## Supplementary Materials

The following supporting information can be downloaded at: https://www.mdpi.com/article/10.3390/ph18010008/s1. References [33,34,35,36,37,38,39,40,41,42,43,44,45,46] are cited in the Supplementary Materials.
