# Supplementary material for: Musa paradisiaca L. Inflorescence Abrogates Neutrophil Activation by Downregulating TLR4/NF-KB Signaling Pathway in LPS-Induced Acute Lung Injury Model
Source: Pharmaceuticals (Basel). 2024 Dec 24;18(1):8. doi: 10.3390/ph18010008 (PMC11768301; doi:10.3390/ph18010008)
Supplement: Supplementary file 1 [file pharmaceuticals-18-00008-s001.zip › pharmaceuticals-3237508-supplementary.pdf]

Material and methods

1. Liquid chromatography coupled with ESI-MSn and HRESIMS analysis

The hydroalcoholic extract and polar fractions of the inflorescence of *Musa paradisiaca* L. were prepared by solubilizing 1.0 mg of each material in 1.0 mL of methanol or 0.5 mL of methanol and 0.5 mL of H_2O. The solutions were injected into a Shimadzu high-performance liquid chromatograph (Kyoto, Japan) equipped with a SIL-20A HT autosampler, two LC-20 AD pumps, a DGU-20A5 online degasser, an SPD-M20A photodiode array detector and a CBM-20A control system, which was coupled to an Amazon X ion-trap mass spectrometer (Bruker Daltonics, Billerica, MA, USA) with an electrospray ionization source. To perform ESI-MSn analysis in negative ionization mode, the capillary voltage was adjusted to 4.5 kV, and the temperature was set to 300 ºC. The drying gas flow rate (N^2) was 8 mL/min, and the nebulizer pressure was 40 psi. CID fragmentation on the Amazon X was performed in automatic MS^n mode using the enhanced resolution mode for MS and MS/MS acquisitions.

Concerning the liquid chromatography system, ultra-purified acidified water (0.1% formic acid) and HPLC grade methanol were used as mobile phases A and B, respectively. For the elution method, the concentration of mobile phase B changed from 5% to 100% in a 60-minute run, and the flow rate was 0.6 mL/min. Chromatographic separation was performed on a Kromasil C18 (250 mm × 4.6 mm, particle size of 5 μm) analytical column (Bohus, Sweden).

Posteriorly, these samples were again injected into a high-performance liquid chromatograph using the same method as previously reported, but this time, the high-performance liquid chromatograph was coupled to a micrOTOF II mass spectrometer (Bruker Daltonics, Billerica, MA, USA) for HRESIMS analyses. The compounds were tentatively identified by the interpretation of their fragmentation patterns (MS^2 and MS^3) and correlations of their corresponding data with scientific literature.

Results

The data from the HPLC-MS/MS are described in Figure S1 and Table S1.

The base peak chromatograms (BPC) of the hydroalcoholic extract (A) were obtained by HRESIMS analysis in order to obtain the chemical profile by comparing the molecular formula and retention time of the compounds that were dereplicated from the ethyl acetate (B) and n-butanolic (C) fractions of *Musa paradisiaca* L inflorescence. The analysis was carried out using HPLC-MS and each number corresponds to the substance described immediately below the chromatograms and is chemically described in Table S1.

Figure S1. Identification of compounds in the hydroalcoholic extract (A) and polar fractions ethyl acetate (B) and n-butanol (C) of the inflorescence of *Musa paradisiaca* L.

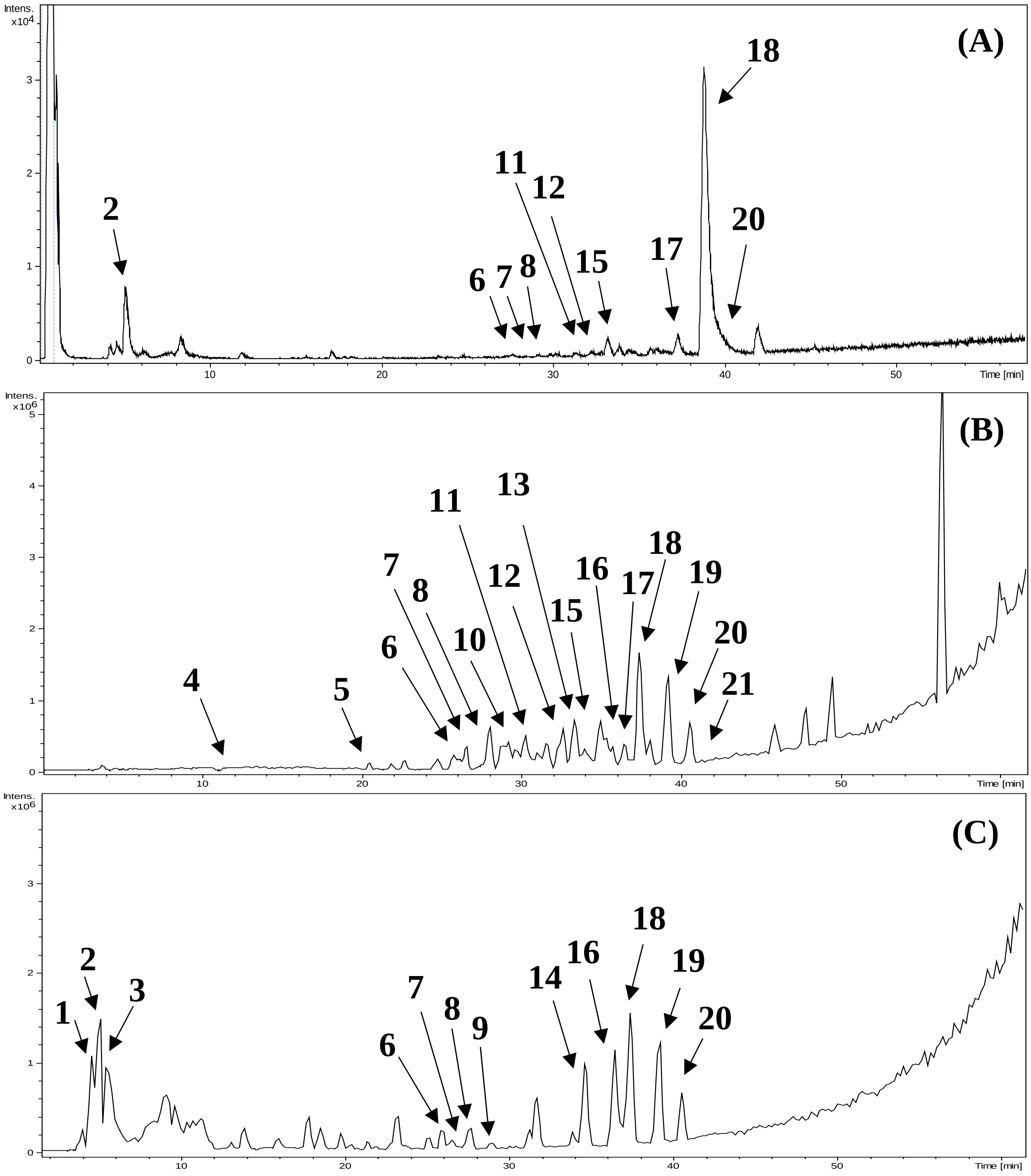

Table S1. Characterization and peak annotation of the compounds presented on ethyl acetate and n-butanolic fraction of *Musa paradisiaca* L. extract by HPLC-MS/MS methodology.

N°	RT (min)	[M-H]$^-$	MS^2, MS^3	EEBM	FAc	Fn-But	Putative identification	Reference
1	5.2	533	MS^2 [533]: 191 (100); MS^3 [533 →191]: 173 (22), 153 (11), 127 (45), 111 (27), 93 (100), 85 (61)	-	-	+	Quinic acid hexoside	1
2	5.3	191	MS^2 [191]: 173 (98); 127 (100); MS^3 [191 → 173]: 161 (37), 150 (15), 143 (100), 131 (19), 119 (7), 107 (22), 89 (19); MS^3 [191 → 127]: 109 (67), 99 (90), 85 (100), 57 (12)	+	-	+	Quinic acid	1
3	5.4	377	MS^2 [377]: 341 (78); 179 (100); MS^3 [377 → 179]: 161 (89), 143 (100), 131 (29), 125 (23), 119 (41), 113 (16), 89 (32), 71 (13)	-	-	+	Disaccharide (HCl adduct)	2
4	14.5	169	MS^2 [169]: 125 (100); MS^3 [169→125]: 97 (78), 81 (100)	-	+	-	Gallic acid	3
5	20.7	153	MS^2 [153]: 109 (100); MS^3 [153 →109]: 107 (100)	-	+	-	Protocatechuic acid	4,5
6	27.4	571	MS^2 [571]: 529 (100), 511 (21), 435 (50), 341 (7), 307 (34); MS^3 [571 → 529]: 505 (24), 487 (100), 469 (25), 307 (25), 236 (21)	+	+	+	di-O-acetyl-p-coumaroylsucrose isomers I	14
7	29.0	571	MS^2 [571]: 529 (100), 511 (36); MS^3 [571 → 529]: 487 (100), 469 (11), 413 (4), 376 (4), 341, 307 (8); MS^3 [571 → 511]: 424 (58), 307 (100), 276 (54)	+	+	+	di-O-acetyl-p-coumaroylsucrose isomers II	14
8	29.3	571	MS^2 [571]: 529 (100); 511 (38); MS^3 [571 → 529]: 487 (65), 460 (100), 442 (95), 410 (42); 376 (74), 354 (23), 258 (78); MS^3 [571 → 511]: 487 (100), 349 (59), 307 (64)	+	+	+	di-O-acetyl-p-coumaroylsucrose isomers III	14
9	29.6	335	MS^2 [335]: 179 (100); MS^3 [335 →179]: 135 (100)	-	-	+	4-*O*-caffeoylshikimic acid I	6
10	30.7	197	MS^2 [197]: 169 (75), 125 (11); MS^3 [197 →169]: 97 (37), 81 (43)	-	+	-	Ethyl gallate	7

11	32.4	613	MS^2 [613]: 571 (100), 553 (39), 511 (14), 476 (15), 426 (3), 383 (7), 350 (4), 307 (17), 277 (3); MS^3 [613 → 571]: 529 (100), 425 (68); MS^3 [613 → 553]: 349	+	+	-	tri-O-acetyl-3-O-p-coumaroylsucrose isomers I	14
12	33.3	613	MS^2 [613]: 571 (100), 553 (73), 529 (18), 511 (14), 409 (3), 384 (3), 349 (6), 307 (7); MS^3 [613 → 571]: 529 (100), 512 (47); MS^3 [613 → 553]: 511 (100)	+	+	-	tri-O-acetyl-3-O-p-coumaroylsucrose isomers II	14
13	33.9	463	MS^2 [463]: 301 (100); MS^3 [463 →301]: 255 (16), 229 (8) 201 (29), 179 (55), 151 (100), 121 (6)	-	+	-	Quercetin-hexose	8, 6, 12
14	34.6	625	MS^2 [625]: 316 (100), 317 (59), 271 (25.8); MS^3 [625 → 316]: 271 (100), 179 (17), 151 (8); MS^3 [625 → 271]: 243 (100)	-	-	+	Myricetin rhamno- hexoside	9, 10, 11
15	35.0	613	MS^2 [613]: 571 (100), 553 (68), 529 (37), 425 (4), 383 (7), 341 (4), 307 (3); MS^3 [613 → 571]: 529 (100), 511 (14), 470 (5); MS^3 [613 → 553]: 511 (23), 493 (100)	+	+	-	tri-O-acetyl-3-O-p-coumaroylsucrose isomers III	14
16	36.5	609	MS^2 [609]: 301 (100) MS^3 [609→301] 271 (100), 255 (44), 227 (23), 210 (49), 179 (64), 151 (69), 121 (3)	-	+	+	Rutin	12
17	37.3	655	MS^2 [655]: 529 (100), 511 (26), 435 (18), 383 (8), 307 (40); MS^3 [571 → 529]: 487 (100), 470 (62); MS^3 [571 → 307]: 216 (35), 145 (47)	+	+	-	tetra-O-acetyl-p-coumaroylsucroses isomers	14
18	37.5	723	MS^2 [723]: 678 (100); MS^3 [723→678]: 659 (100), 563 (35), 492 (5), 451 (23), 338 (25)	+	+	+	1-Sinapoyl-2-feruloylgutiobiose	13
19	39.3	836	MS^2 [836]: 791 (100); MS^3 [836→791]: 772 (100), 678 (44), 451 (18), 338 (49)	-	+	+	Hydroxycinnamic acid esters I	13
20	40.6	949	MS^2 [949]: 903 (100); MS^3 [949→903]: 885 (100), 772 (14), 678 (28), 564 (50), 451 (42)	+	+	+	Hydroxycinnamic acid esters II	13
21	43.5	593	MS^2 [593]: 447 (27), 285 (100); MS^3 [593→285] 257 (93), (53), 213 (71), 185 (44), 169 (58), 151 (100), 119 (36)	-	+	-	Kaempferol-O-rutinosídeo	8

The references listed in the Table S1 are described below.

1. SPÍNOLA, V.; PINTO, J.; CASTILHO, P.C. Identification and quantification of phenolic compounds of selected fruits from Madeira Island by HPLC-DAD–ESI-MSn and screening for their antioxidant activity. **Food Chemistry,** v. 173, p. 14 - 30, 2015.

2. ZENGINA, G.; LLORENT-MARTÍNEZB, E. J.; CÓRDOVAB, M. L. F.; BAHADORIC, M. B.; MOCAND, A.; LOCATELLIF, M.; AKTUMSEKA, A. Chemical composition and biological activities of extracts from three Salvia species: *S. blepharochlaena*, *S. euphratica var. leiocalycina*, and *S. verticillate subsp. Amasiaca.* **Industrial Crops & Products,** v. 111, p. 11-21, 2018.

3. LEE, R.; LEE. V. S. Y. ; TZEN, J. T.C.; LEE, M. Study of the release of gallic acid from (–)-epigallocatechin gallate in old oolong tea by mass spectrometry. **Rapid Commun. Mass Spectrom,** v. 24, p- 851-858, 2010.

4. KANG, J.; PRICE, W. E.; ASHTON, J.; TAPSELL, L. C.; JOHNSON, S. Identification and characterization of phenolic compounds in hydromethanolic extracts of sorghum wholegrains by LC-ESI-MSn. **Food Chemistry**, v. 211: p. 215-226, 2016

5. BOUHAFSOUN, A., YILMAZ, M. A.; BOUKELOUA, A.; TEMEL, H.; KAID HARCHE, M. Simultaneous quantification of phenolic acids and flavonoids in *Chamaerops humilis* L. using LC–ESI-MS/MS. **Food Science and Technology,** v. 38, p. 242-247, 2018.

6. DANTAS, C. A. G; ABREU, L. S.; CUNHA, H. N.; VELOSO, C. A. G.; SOUTO, A. L.; AGRA, M. F.; COSTA, V. C. O.; SILVA, M. S.; TAVARES, J. F. Dereplication of phenolic derivatives of three *Erythroxylum* species using liquid chromatography coupled with ESI-MSn and HRESIMS. **Phytochemical Analysis.** 2021;1–16.

7. HE, Z; XIA, W. Analysis of phenolic compounds in Chinese olive (*Canarium album* L.) fruit by RPHPLC–DAD–ESI–MS. **Food Chemistry**, v. 105, p. 1307–1311, 2007.

8. SOUZA, R. B. L.; NASCIMENTO, Y. M, GOUVEIA, R. G.; SOUTO, A. L.; SOBRAL, M. V.; COSTA, V. C. O.; MELO, J. I. M.; SILVA, M. S.; TAVARES,

J. F. Dereplication-guided isolation of a new flavonoid triglycoside from *Macroptilium martii* and its cytotoxicity evaluation. **Phytochemistry Letters**, v. 39, p. 144-150, 2020.

9. BYSTROM, L. M.; LEWIS B. A; BROWN, D. L.; RODRIGUEZ, E; OBENDORF, R. L. Characterisation of phenolics by LC–UV/Vis, LC–MS/MS and sugars by GC in *Melicoccus bijugatus* Jacq. ‘Montgomery’ fruits. **Food Chemistry**, v. 111, p. 1017-1024, 2008

10. DING, S.; DUDLEY, E.; PLUMMER, S.; TANG, J.; NEWTON, R. P.; BRENTON, A. G. Fingerprint profile of *Ginkgo biloba* nutritional supplements by LC/ESI-MS/MS. **Phytochemistry**, v. 69, p. 1555–1564, 2008.

11. RIEHLE, P.; VOLLMER, M.; ROHN, S. Phenolic compounds in *Cistus incanus* herbal infusions — Antioxidant capacity and thermal stability during the brewing process. **Food Research International**, v. 53, p. 891–899, 2013.

12. ENGELS, C.; GRÄTER, D.; ESQUIVEL, P. JIMÉNEZ, V. M. GÄNZLE, M. G.; SCHIEBER, A. Characterization of phenolic compounds in jocote (*Spondias purpurea* L.) peels by ultra high-performance liquid chromatography/electrospray ionization mass spectrometry. **Food Research International,** v. 46, p. 557–562, 2012.

13. DE-MELO, A. A. M.; ESTEVINHO, L. M.; MOREIRA, M. M.; DELERUE-MATOS, C.; FREITAS, A. S.; BARTH, O. M.; ALMEIDA-MURADIAN, L. B. Phenolic profile by HPLC-MS, biological potential, and nutritional value of a promising food: Monofloral bee pollen. **Food Biochem.** 2018.

14. ZHANG, X.; LIN, Z.; FANG, J.; LIU, M.; NIU, Y.; CHEN, S.; WANG, H.; An on-line high-performance liquid chromatography–diode-array detector–electrospray ionization–ion-trap–time-of-flight–mass spectrometry–total antioxidant capacity detection system applying two antioxidant methods for activity evaluation of the edible flowers from *Prunus mume.* **Journal of Chromatography A.** 2015.
